# Forward suppression in the auditory cortex is frequency-specific

**DOI:** 10.1111/j.1460-9568.2010.07568.x

**Published:** 2011-04

**Authors:** Chris Scholes, Alan R Palmer, Christian J Sumner

**Affiliations:** MRC Institute of Hearing Research, University ParkNottingham NG7 2RD, UK

**Keywords:** auditory cortex, context dependence, forward suppression, frequency specificity, guinea pig

## Abstract

We investigated how physiologically observed forward suppression interacts with stimulus frequency in neuronal responses in the guinea pig auditory cortex. The temporal order and frequency proximity of sounds influence both their perception and neuronal responses. Psychophysically, preceding sounds (conditioners) can make successive sounds (probes) harder to hear. These effects are larger when the two sounds are spectrally similar. Physiological forward suppression is usually maximal for conditioner tones near to a unit's characteristic frequency (CF), the frequency to which a neuron is most sensitive. However, in most physiological studies, the frequency of the probe tone and CF are identical, so the role of unit CF and probe frequency cannot be distinguished. Here, we systemically varied the frequency of the probe tone, and found that the tuning of suppression was often more closely related to the frequency of the probe tone than to the unit's CF, i.e. suppressed tuning was specific to probe frequency. This relationship was maintained for all measured gaps between the conditioner and the probe tones. However, when the probe frequency and CF were similar, CF tended to determine suppressed tuning. In addition, the bandwidth of suppression was slightly wider for off-CF probes. Changes in tuning were also reflected in the firing rate in response to probe tones, which was maximally reduced when probe and conditioner tones were matched in frequency. These data are consistent with the idea that cortical neurons receive convergent inputs with a wide range of tuning properties that can adapt independently.

## Introduction

The responses of auditory neurons decrease over time with continuous or repeated stimulation. This ‘adaptation’ can be considered as a mechanism to provide sensitivity for the stimulus context by suppressing responses to ongoing stimuli. The auditory system is sensitive to recent stimulus history along a number of dimensions (Malone & Semple, 2001; Ulanovsky *et al.*, 2003; Ingham & McAlpine, 2004; Nakamoto *et al.*, 2006; Scholl *et al.*, 2008). This may serve to maintain the tuning of neurons at a near optimal point (Dean *et al.*, 2005) or to emphasize new and interesting sounds (Ulanovsky *et al.*, 2003; Malmierca *et al.*, 2009). Adaptation may also underlie perceptual phenomena, such as the organization of streams of sounds (Fishman *et al.*, 2004) or the masking of weak signals by preceding sounds (Plomp, 1964; Relkin & Turner, 1988; Nelson *et al.*, 2009).

The adaptation of neural responses has been investigated at various levels of the auditory system using two-tone sequences (Harris & Dallos, 1979; Calford & Semple, 1995; Brosch & Schreiner, 1997; Bleeck *et al.*, 2006). Generally, the response to a second tone (probe) is reduced when preceded by a tone (conditioner) of a similar frequency. We will refer to this as forward suppression. In auditory nerve fibres, the reduction in the response to the probe tone is proportional to the number of spikes evoked by the conditioner tone (Smith, 1977; Harris & Dallos, 1979). Hence, the tuning of forward suppression closely resembles the single-tone excitatory receptive field (RF; Harris & Dallos, 1979). However, in central auditory neurons forward suppression can also depend on effects such as inhibition (e.g. Shore, 1998) and considerable below-threshold processing of the neuronal inputs occurs, which is not evident in spiking activity (Xie *et al.*, 2007). Preceding sounds can also lead to facilitation of responses (Brosch & Schreiner, 2000). Thus, in cortical neurons, the tuning of the forward suppression is often qualitatively different from the excitatory RF and not well accounted for by preceding spiking activity (Calford & Semple, 1995; Brosch & Schreiner, 1997).

Previous studies of forward suppression using two tones have concentrated on the case when the probe tone is at the characteristic frequency (CF) of the neuron (Harris & Dallos, 1979; Boettcher *et al.*, 1990; Brosch & Schreiner, 1997). In this case, it is not possible to disambiguate CF-specific suppression from frequency-specific suppression. One study that did use off-CF probe tones (in seven neurons) concluded that cortical forward suppression was ‘biased by the probe frequency rather than centred on it’ (Calford & Semple, 1995). In the inferior colliculus (IC) there is also some evidence that setting the probe frequency away from CF affects the tuning of suppression (Malone & Semple, 2001). This may depend on the location within the central nucleus of the IC (Stakhovskaya *et al.*, 2008).

As it remains unclear whether forward suppression in the cortex is determined by the probe frequency or the tuning properties of the neuron, we investigated systematically the extent to which forward suppression in cortical neurons is specific to the frequency of the probe in a two-tone sequence.

## Materials and methods

### Animal preparation

Experiments were performed on 24 pigmented guinea pigs (which also contributed data to other studies) of both sexes that weighed 325–813 g (mean 573 g). Each guinea pig was anaesthetized with an intra-peritoneal injection of urethane (4.5 mL/kg in a 20% solution) supplemented with intra-muscular injections of 0.2 mL Hypnorm (fentanyl citrate 0.315 mg/mL, fluanisone 10 mg/mL) whenever a forepaw withdrawal reflex could be elicited. A pre-medication of 0.2 mL atropine sulphate (600 mu/mL) was administered subcutaneously to suppress bronchial secretions. Each animal was tracheotomized, artificially respired and rectal temperature was maintained at 38 °C by means of a heating blanket. The animals were placed in a stereotaxic frame with hollow plastic specula replacing the ear bars, inside a sound-attenuating room. To equalize pressure across the tympanic membrane, the bulla on each side was vented with a polyethylene tube (22 cm long, 0.5 mm diameter), and the membrane overlying the foramen magnum was opened to release the pressure of the cerebrospinal fluid. A craniotomy with a diameter of about 5 mm was performed to expose the primary auditory cortex (A1), the dura was removed and the brain was covered with a layer of Agar. A linear multi-electrode array, consisting of four to eight glass-coated, sharp tungsten micro-electrodes was advanced together into A1 by a piezoelectric motor (Burleigh Inchworm IW-700/710). All experiments were performed in accordance with UK Home Office regulations.

### Acoustic stimuli and electrophysiological recording

Auditory stimuli were delivered diotically through sealed acoustic systems, consisting of modified Radio Shack 40-1377 tweeters coupled to damped probe tubes that fitted into the specula. The system was calibrated a few millimetres from the eardrum by a 1-mm probe tube that was attached to a microphone (Brüel & Kjaer 4134). This was to ensure that sound levels were consistent across experiments (± 3 dB). All stimuli were generated by an array processor (TDT AP2, Alachua, FL, USA) and output at a sample rate of 100 kHz. Stimulus control was from a PC using Brainware (developed by J. Schnupp, University of Oxford). Responses from the electrodes were acquired using a Medusa Headstage and Tucker Davis RX7, sampled at 25 kHz with 16-bit resolution, and digitally filtered (300 Hz–3 kHz) and amplified (approximately ×40 k). Spike waveforms and spike times were recorded to disk by Brainware. They were further analysed off-line with Plexon (Dallas, TX, USA) software to isolate action potentials from separate single units (SU) and/or multi-units (MU). The off-line sorting allowed for supervised spike sorting on the basis of a principal components analysis, as well as on other spike waveform features. SUs were identified as those whose feature vectors formed clear, non-overlapping clusters in feature space (normally the first two principal components) and maintained this distinction for the duration of the recording. Such units did not generate action potentials at inter-spike intervals shorter than 1 ms.

One-hundred-millisecond broadband noise bursts were presented as search stimuli. Once a neuron was located, single 50-ms gated tones (2 ms rise/fall cosine squared ramps) were presented at a rate of 1/s at a range of frequencies and levels in order to determine the RF. The frequencies and levels of two or more tones were selected with reference to the RF, to be used as probe tones. Typically one probe tone frequency was chosen near to, but not at, unit CF, and another was chosen to be distant from CF. Frequently, probes were chosen to be within the RFs of several units simultaneously recorded on different electrodes. To investigate the effect of prior stimulation on the response to the probe tones they were preceded by a conditioner tone. The conditioner tones were varied over a range of frequencies and levels (e.g. 1/4 octave and 10-dB steps). In many cases, there was no temporal gap between the offset of the conditioner and the onset of the probe tone. In other cases, the delay to the probe tone (the interstimulus interval; ISI) was also varied. Typically, ISIs were varied up to 300 ms in steps of 50 or 100 ms. The range and spacing of conditioner tone frequencies and levels, the number of probe tones and the number of repetitions of each combination varied. A single repetition of the entire set of stimulus conditions to be tested in a unit was presented in a completely random (pseudo-random) order. A new pseudo-random order was generated for each repetition of the set, and this was usually repeated three or more times. The entire set of stimuli tested in a unit took between 30 and 60 min to complete. Both the probe tone and the conditioner tones were gated 50-ms tones (2 ms rise/fall cosine squared ramps) with a duty cycle of 1 s.

### RF analysis

The number of spikes elicited by a tone was calculated within a time-window that was fixed for each unit. The window was calculated based on the summed PSTH of the responses to the conditioner across all of the stimulus conditions, and defined as the epoch within which the firing rate in response to the conditioner tone exceeded one standard deviation above the spontaneous rate (SR) of the unit. The window length was limited to a maximum of 50 ms, to avoid counting any response to the probe. The SR was estimated from the last recorded 50 ms of every repetition interval (which was usually 800 ms starting at the onset of the conditioning tones). A RF was then created from the responses to the conditioner tones. An analysis window of the same duration was used to calculate the response to the probe, applied relative to the beginning of the probe stimulus. The resulting suppressed RFs (SRF) described the response to each probe condition as a function of the conditioner tone frequency and level. We only analysed probe conditions in which, when the conditioner was maximally attenuated, the response to the probe was significantly above the SR (*P* < 0.05, bootstrap test described below). We further checked that the response to the maximally attenuated conditioner was not significantly above the SR (*P* > 0.05, bootstrap test described below; 5 units were excluded).

In some analyses, the measure of interest was the firing rates derived as above. However, a major goal of this paper was to assess the effect of probe condition on the tuning of suppression. For these analyses, RF characteristics were extracted automatically. The RFs were smoothed using a pyramidal 3 × 3 window (the product of two triangular windows along the two axes; see Moshitch *et al.*, 2006). Then, iso-response curves were derived, using an automatic algorithm (Sutter & Schreiner, 1991; Moshitch *et al.*, 2006). For the RF, each frequency/level combination was tested to see if the spike rate was larger than the baseline response + criterion value *(maximal response−baseline response), where both instances of the baseline response were the same value: an average of all conditioner tone responses at the lowest presented level (i.e. below threshold). For the SRF, the response to a frequency/threshold combination was considered to have been suppressed if the spike rate was lower than the baseline response−criterion value* (*baseline response−spontaneous response*), where the baseline response was the mean response to the probe tone when it was preceded by conditioner tones presented at the lowest intensity. Two different criterion values were used: a 0.4 criterion traced an iso-response curve near to the edge of the RF or SRF, and defined a frequency threshold curve (FTC) or suppression frequency threshold curve (SFTC); a 0.9 criterion traced out the region of maximal response in the RF or suppression in the SRF. These iso-response curves were each fitted with a 12th-order polynomial function.

The CF of the unit was defined as the frequency at which the lowest threshold occurred for the FTC (i.e. the 0.4 criterion iso-response curve in the RF). The unit best frequency (BF), or the frequency that produced the maximum firing rate, was defined by the frequency with the lowest threshold in the 0.9 criterion iso-response curve. In a similar manner, the suppressed CF (SCF; the frequency that was most sensitive to suppression) was defined from the SFTC (0.4 criterion iso-response curve), and the suppressed BF (SBF; the frequency that produced the most suppression) from the 0.9 criterion iso-response curve. The bandwidth of resulting FTCs and SFTCs was also assessed, using the equivalent rectangular bandwidth (ERB) measure (Moore, 2004). ERBs give the bandwidth of a perfectly rectangular filter that passes the same energy as the measured filter shape. It is widely used in psychophysics and in several physiological studies (Evans, 2001; Sayles & Winter, 2010; Shera *et al.*, 2010) as a measure of the spectral integration properties of a filter. As it takes into account the entire tuning curve, it is not as sensitive to local differences at arbitrary points on a tuning curve, as are Q10s. Q-factors were then derived from the ERBs as CF/ERB (or SCF/ERB), to yield a Q_ERB_.

### Bootstrap statistics of SRFs and tuning

Probe tones with frequencies away from CF often elicited lower and less reliable spiking, and therefore potentially less reliable measures of suppressed tuning. In order to assess the reliability of the suppressed tuning, we employed a bootstrap method that takes into account the spiking statistics of the individual neuron. Any given combination of conditioner (frequency/level) and probe (frequency, level, ISI) presented four times would yield four spike counts (e.g. 2, 3, 0 and 2 spikes on four different presentations) in response to the probe tone. This is not enough to generate any reliable estimates of response variability, or to assess the accuracy of our estimate of the underlying mean firing rate. Instead, we calculated the probability that this combination of spikes would occur for a range of underlying mean firing rates (e.g. 2, 2.25, 2.5, etc. spikes/presentation). The spiking statistics for the mean firing rates were estimated from the response to the conditioner, for which there were many more presentations for each condition (repeated for every probe). Of course, this assumes that the spiking statistics in response to the conditioner and probe tone were the same. Whilst this assumption cannot be confirmed from these data, we have shown in another study that the mean rate and variance of firing in cortical neurons is closely related, irrespective of the combination of masker and probe level (Alves-Pinto *et al.*, 2010).

First, we characterized the spiking statistics of a unit as a function of the mean firing rate in response to the conditioner. Every conditioner frequency/level combination yielded a mean firing rate. For a given mean firing rate (grouped in intervals of 0.25 spikes/presentation), irrespective of the actual stimulus condition, we constructed a histogram of spike counts to individual stimulus presentations. Dividing by the number of stimulus presentations yielded a distribution describing the probability of recording a given number of spikes on any given stimulus presentation, for a known mean rate. Next, for each measured response to the probe, for a given probe/conditioner combination, we used this spike count distribution to estimate the likelihood of different underlying mean firing rates. Thus, we arrived at another distribution for that particular probe response, which described the range and probability of likely underlying mean rates, based on the actual spike count statistics derived from the response to the conditioner tones.

Given a probability distribution of underlying mean firing rates for each probe response, we can use this to simulate many re-runs of collecting the same data, thus generating bootstrapped statistics. For every probe condition and every conditioner frequency/level combination, we re-estimated (by randomly sampling from the calculated probability distribution) the mean firing rate, and then performed the same data analysis described above to generate SCFs, SBFs and their associated thresholds. This was repeated 500 times for every probe condition. This yielded statistical distributions of the quantities derived from the RFs (standard deviations, 95% confidence bounds) and new estimates of the mean values, which were used for the analysis of tuning. Thus, for any measure, for example SCF, threshold and Q_ERB_, we were able to estimate the reliability.

### Population statistics

**Fig. 3:**
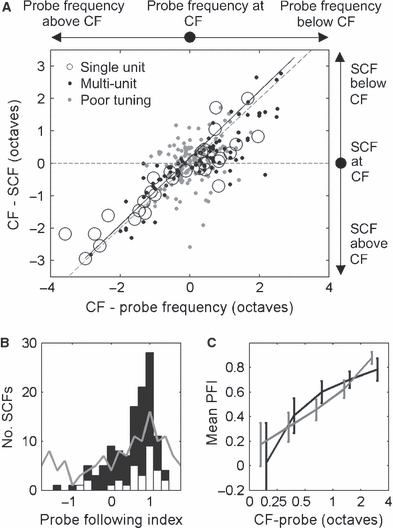
Variation of the suppressed characteristic frequency (SCF) with the probe frequency across the population, for an ISI of zero. (A) The distance between the probe and the characteristic frequency (CF) in octaves is shown on the abscissa, and the distance between the SCF and the CF in octaves is shown on the ordinate for SUs (open circles) and MUs (black points). Grey points indicate data where SCF was less reliably estimated. (B) Histogram showing the probe-following index (PFI) of the SCF for SUs (open bars) and MUs (black bars). Grey line shows the PFI for less reliably estimated SCFs. (C) The variation in PFI for probe frequencies at different distances away from CF (black lines show SU and MU data where SCF was accurately measured; grey lines show data where SCF was less reliable).

**Fig. 4:**
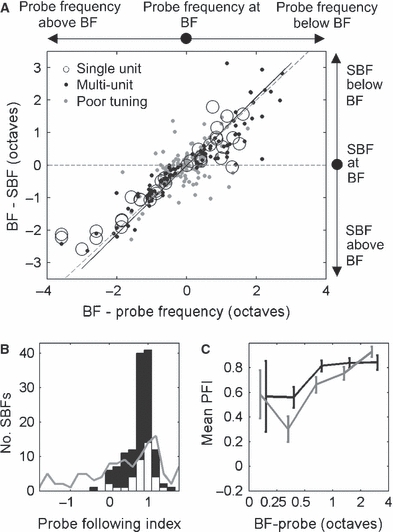
Variation of the suppressed best frequency (SBF) with the probe frequency across the population, for an ISI of zero. (A) The distance between the probe and the best frequency (BF) in octaves is shown on the abscissa, and the distance between the SBF and the BF in octaves is shown on the ordinate for SUs (open circles) and MUs (black points). Grey points indicate data where SBF was less reliably estimated. (B and C) Probe-following index (PFI) derived from SBF and BF, as per Fig. 3.

To characterize the locus of suppression (SCF for the tuning of suppression near to threshold or SBF for the conditioner frequency that suppresses most effectively) relative to the single-tone excitatory CF (or BF) and the probe tone frequency, a probe-following index (PFI) was calculated. This characterizes the angle that each data point forms with the horizontal axis of the representation shown in Figs 3 and 4. In the figures, the difference between unit CF and the SCF in octaves [log_2_(CF/SCF)] is plotted against the difference between the frequency of the probe and CF [log_2_(CF/F_probe_)]. The PFI for the SCF is given by: 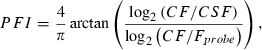
(1) where F_probe_ is the frequency of the probe. A value of 1 corresponds to points that followed the probe tone frequency perfectly. In such cases SCF = F_probe_, and points lie on the ascending diagonal in the figures. A value of zero indicates that the SCF was identical to the CF (the numerator in Eq. 1 is then a log of 1, which is 0). For the calculation of the PFI associated with the region of maximal suppression, the BF and SBF measures were used.

We also characterized the frequency specificity of suppression in terms of firing rate, in an analysis similar to that employed by Ulanovsky *et al.* (2003, 2004). We considered pairs of probe conditions within a unit, p_1_ and p_2_, and for each probe condition we considered the effect of conditioners, c_1_ and c_2_, at both of those frequencies. Thus, for p_1_ we were interested in the firing rates r(p_1_|c_1_), the firing rate in response to a probe at frequency 1 in the presence of a conditioner at frequency 1, and r(p_1_|c_2_). Because for each combination of probe and conditioner, the conditioner level was varied, we have a pair of rate-level functions of the conditioner level. If the firing rates in response to the probes are lower when the probes and conditioners are matched in frequency, then suppression is to some degree frequency specific. To generate a summary statistic of this, we considered the firing rate summed across conditioner level. The degree to which suppression of the probe condition p_1_ relies on the conditioner being matched is then given by a specificity index (SI): 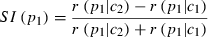
(2)

This yields a number between −1 and 1. If the rate response to p_1_ is more suppressed when preceded by a conditioner of matched frequency c_1_, then the number will be positive. If c_2_ produces more suppression the number will be negative. A similar value can be computed for p_2_. A single overall value for a unit was also calculated similar to Ulanovsky *et al.* (2003, 2004), by combining across all pair combinations at a given ISI. Thus: 
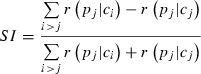
(3) where r(p_j_|c_i_) is the firing rate in response to probe *j* in the presence of conditioner *i*.

### Bootstrap significance test for spike counts

Trial-by-trial comparisons of evoked firing rates, often with spontaneous activity, were made using bootstrap methods to predict the probability that two populations with the measured difference in means could arise if the two groups belonged to the same underlying population. The samples from both original populations were pooled, and then repeatedly randomly redistributed into two groups (500 times). For each resampling, the mean difference between the two groups was calculated. This generated a distribution of differences for the means, and allowed us to estimate the probability (our *P*-value) that these two groups could have the observed difference in the mean if the samples were drawn from a single population.

## Results

### The influence of probe tone frequency on SRFs

**Fig. 1:**
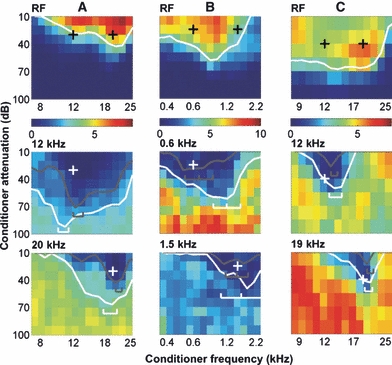
Examples of the shift in forward suppression with different probe frequencies. Top row shows single tone excitatory receptive fields (RFs), threshold (solid white line) FTCs and the probe conditions (black crosses). Remaining rows show the suppressed RF (SRF) for the different probe tones. (A) SU example, where a 12-kHz or 20-kHz probe tone is preceded by conditioner tones at the frequencies and levels indicated on the axes. The colour bar indicates the number of spikes elicited per stimulus presentation. SFTCs are indicated by solid white lines, and grey lines indicate regions of maximal suppression. The bars below different tuning curves indicate the mean SCF/SBF (middle tick; this is omitted for clarity in some panels) and the standard deviation of their estimate (left and right ticks), derived from the bootstrapping. The probe condition is indicated by a white cross. (B and C) SU examples as per (A).

A total of 156 units were recorded (53 SU and 103 MU). Figure 1 shows several example units where we measured SRFs for several different probe tone frequencies. In these examples the probe immediately follows the conditioning tone (i.e. an ISI of 0). The first row shows the excitatory RF of each unit and beneath it are the SRFs for each probe condition. Figure 1A shows a SU in which the tuning of suppression is profoundly affected by the choice of probe. In this example, two probe tone frequencies were presented (indicated by crosses): one at 20 kHz, close to the CF; and one at 12 kHz, away from the CF. When the probe frequency was 20 kHz, near to the unit CF, the SRF had a similar shape to the RF, and both the SCF and SBF are at the probe tone frequency. When the probe tone frequency was at 12 kHz, well below the unit's CF, the SRF shifted to lower frequencies. The tuning near threshold (0.4 criterion; white line) and near maximum suppression (0.9 criterion; grey line) clearly showed a shift in tuning. Suppression also had a lower threshold and a wider bandwidth for the off-CF probe. Two further examples are given in Fig. 1, both of which show differences in the tuning of forward suppression when the probe tone frequency is not at CF. Notice that the tuning is not necessarily centred on the probe frequency. In some cases, the region of maximal suppression (grey lines) is centred on the probe frequency whilst threshold tuning resembles the excitatory tuning curve (Fig. 1B; 0.6-kHz probe).

**Fig. 2:**
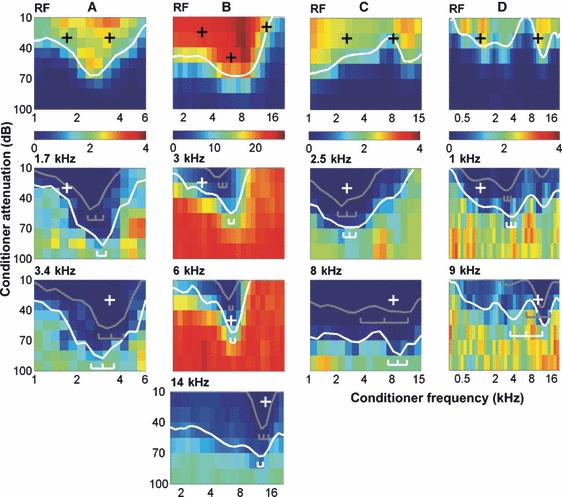
Four examples of forward suppression for different probe frequencies. Same conventions as Fig. 1. (A) SU example where there is little effect of the probe frequency. (B) MU example where three different probe frequencies were presented. (C) SU example of the effect of varying the probe frequency in a complex tuning curve. (D) Another example of a SU with a multi-peaked excitatory tuning curve. RF, receptive field.

Four more examples are displayed in Fig. 2. The example shown in Fig. 2A demonstrates that the suppression was not always strongly influenced by the probe tone frequency. For both probe tones, the SCF and SBF are closer to the CF than to the probe tone frequency. There are, however, differences between the SRFs for the two probe tone frequencies. The 1.7-kHz (below CF) probe tone is less strongly suppressed by above CF tones than when the probe is at 3.4 kHz (above CF). In the example shown in Fig. 2B, there is only a subtle expansion of the tuning curve towards the probe frequency for a probe frequency below CF (Fig. 2B; 3-kHz probe), compared with when the probe tone frequency is closer to unit CF; whereas, when the probe is above CF (14 kHz) there is a clear shift in the SRF.

**Fig. 5:**
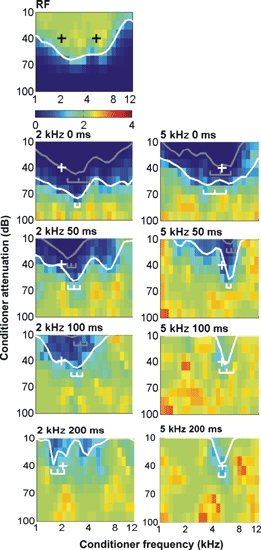
SU example of suppression as the ISI is varied with two different probe tone frequencies. The ISI is indicated above each SRF. Left column: the receptive field (RF) and SRFs for the 2-kHz probe condition. Right column: the SRFs for the 5-kHz probe condition. Each panel shows FTCs or SFTCs (white lines), regions of maximum masking (grey lines), probe conditions (black and white crosses), and bootstrapped mean and standard deviations of SCF and SBF, as per Figs 1 and 2.

The example shown in Fig. 2C had multiple peaks in the excitatory RF. In each condition, the SCF and SBF tend towards the probe tone frequency. However, the range of conditioner tones that suppress the 8-kHz probe tone is larger than the range of conditioner tones that suppress the 2.5-kHz probe tone. There is also some suggestion that suppression for the 8-kHz probe is centred on the upper lobe in the excitatory RF, at 10 kHz. Clear multi-peaked tuning curves were relatively rare in our sample (12 units). While it was clear that suppressed tuning was biased towards the probe tone, there was frequently evidence that local features in the RF, near to the probe frequency, also had an influence on tuning. Figure 2D shows another example of a multi-peaked tuning curve in which suppression tended to gravitate towards the peak in the excitatory tuning curve that was nearest to the probe frequency (see also Fig. 5).

### The locus of tuning for suppression

It is clear from Figs 1 and 2 that the choice of probe tone frequency affects the measured SRF of a unit, and that the general effect is often a shift of the suppressed tuning in the direction of the probe tone frequency. Although this was clearly more complicated than a simple shift in the tuning curve, we sought to evaluate the degree to which SRFs across the population were tuned to either the probe frequency or the excitatory CF. We evaluated this first for conditions where the probe immediately followed the masker. Figure 3A displays the distance from the CF to the SCF in octaves as a function of the distance from the probe tone frequency to the SCF. Lines indicate the trend that would be expected if the SCF was always at the frequency of the CF (horizontal grey dashed line) or at the probe tone frequency (diagonal grey dashed line). Many of the data lie close to the diagonal, indicating a tendency for tuning to follow the probe tone frequency rather than the unit CF. A linear regression against all the data shown in Fig. 3A yields a significant correlation and a gradient of 0.73 (*R* = 0.69, *P* < 0.0001). This suggests that SCFs are biased substantially towards the probe tone frequency.

A number of factors could influence the observed relationship between the probe frequency and the tuning of forward suppression. In particular, probes with frequencies away from CF could elicit lower, more variable spike counts (also reported in Brosch & Schreiner, 2000) than probes at CF (this is explored further below). We reasoned that this would affect the reliability of our tuning estimates. Additionally, probe tone frequencies near to CF cannot provide any useful test of the hypothesis that the frequency of the probe tone affects the tuning of suppression (they should sit near the origin in Fig. 3A). We wished to see if the relationship between probe frequency and suppressed tuning was any different if we considered separately the most reliable data, and only those far enough away from CF to provide a good test of the hypothesis. Figure 3A shows in black the SU (open circles) and MU (points) data for which the standard deviation of the SCF estimate was less than half the distance between the probe and the CF (i.e. [CF − probe] was more than twice the standard deviation of the SCF estimate). These data show a tighter clustering around the diagonal than do the grey points. A linear regression of the SU and MU data for which reliable estimates of SCF were obtained is displayed as a solid black line. A gradient of 1.03 with *R* = 0.88, *P* < 0.0001 (for SUs alone: *m* = 1.07, *R* = 0.91, *P* < 0.00001) indicates that the SCF was closely aligned to the probe tone frequency. The distance from the SCF to the CF was not found to be significantly different for the SU (open circles) and MU (black points) populations (one-way anova, *P* > 0.05).

To summarize the degree to which the SCF corresponded to the probe frequency (i.e. lay on the diagonal in Fig. 3A) or the SCF corresponded to the CF (i.e. lay on the horizontal line in Fig. 3A) in each condition, we computed a PFI (see Materials and methods). The trend for suppression to be tuned to the probe frequency is also clear in the PFI values shown in Fig. 3B, for SU (open bars) and MU (black bars) data where SCFs were accurately determined. The majority of SCFs form a skewed distribution with peak near to the PFI value of 1, indicating that suppression is generally biased towards the probe tone frequency, but with a tendency for SCFs to lie between the CF and the probe frequency. In Fig. 3C the PFI is plotted against the frequency difference in octaves between the probe tone frequency and CF. The data show a tendency for SCF to be closer to the probe frequency when it is further from CF (regression against individual PFI values for the reliably estimated SCFs: *R* = 0.57, *P* < 0.0001; black line in Fig. 3C). Nevertheless, the mean PFI is significantly different from zero for all probe frequency groups (*t*-tests: *P* < 0.01 in all groups except for probe frequencies <0.25 octaves from CF). Even considering all the data the same trend is evident (grey line).

It was clear that the accuracy of SCF estimation contributed to the scatter in Fig. 3A. It was possible that this might reflect some interesting feature of the neuron, such as a change in the statistics of suppression resulting from stimulating weaker synapses not tuned to CF. However, several other factors were likely to affect this accuracy. Most importantly, if the spike count elicited by the probe when there was no conditioner was small or unreliable, then the tuning of suppression would be poorly estimated. This was expected as a consequence of choosing probe conditions away from CF. The bandwidth of tuning might also affect accuracy, as any variability would shift the minima around more if the slopes of the tuning curves were shallower. Consistent with this, the bootstrapped standard deviations of the individual SCF estimates (expressed in octaves) correlated significantly with the mean spike rate in response to the probe alone (normalized to the maximum rate in the excitatory RF; *R* = 0.32, *P* < 0.0001), the probability of there being no spike at all on any given presentation of the probe (*R* = 0.39, *P* < 0.0001), and the quality factor of the bandwidth of the suppressed tuning (Q_ERB_; see Materials and methods; *R* = 0.39, *P* < 0.0001). A linear regression against all three variables was found to account for 59% (*P* < 0.0001) of the variance in the SCF. Thus, the variability of the SCF was in large part a consequence of the reliability of the response to the probe, and was also related to the bandwidth of suppressed tuning.

Figure 4 is similar to Fig. 3, except that the BF and SBF (near maximal suppression) are displayed. The linear regression for the more reliably estimated SBFs (*m* = 1.05, *R* = 0.93, *P* < 0.0001) shows that more of the variance is accounted for by the fit than for the SCF, and the SBF was generally closer to the probe tone frequency. This was also true if we considered all of the data (*m* = 0.96, *R* = 0.86, *P* < 0.0001). Again, the distance between the SBF and the BF for the SU and MU populations was not found to be significantly different (one-way anova, *P* = 0.94). Figure 4B displays a histogram of the PFI for the SBF. Thus, the region of maximal suppression is highly frequency specific in many conditions, whilst the tuning as evidenced by the threshold of suppression (Fig. 3) is less tightly linked to the choice of probe tone frequency. There is a less marked relationship between the PFI for the maximal suppression and the distance between the CF and the probe, consistent with the tighter range of PFIs in Fig. 4B.

One other question that we addressed was whether units formed distinct sub-populations with different behaviour with respect to probe tone frequencies. In 34 units two or more probe tones were presented at frequencies away from CF that exceeded the accuracy of the SCF determination by two standard deviations (as in Fig. 3). Of these units, in two units PFIs were not significantly different from zero (*P* < 0.05; from bootstrapped PFI distribution) irrespective of the probe tone frequency. Sixteen (47%) units had PFIs significantly different (PFI > 0, *P* < 0.05) from zero in the direction of the probe tone frequency in all probe conditions. Thus, approximately half of the units showed frequency specificity of forward suppression in all conditions, and half of the units (16 units, 47%) showed a mix of effects, with some probe tone frequencies resulting in a significant bias of suppression towards them and others not.

### The locus of suppressed tuning when the temporal gap between the conditioner and probe was varied

In 82 units (30 SU, 52 MU) we also recorded responses to the probe with different ISIs between conditioner and probe tone. The trend observed with no gap for the SCF and SBF data is maintained when the gap between the conditioner and probe tone is increased. This can be seen in the SU example shown in Fig. 5. As ISI increases, the suppressed bandwidth decreases and threshold increases, with suppression receding towards each probe tone frequency. Also notice, however, that different features in the excitatory RF appear to influence suppressed tuning in each probe condition. In some cases suppressed tuning is matched to these features in the excitatory RF that are close to the probe frequency, rather than probe frequency itself.

**Fig. 6:**
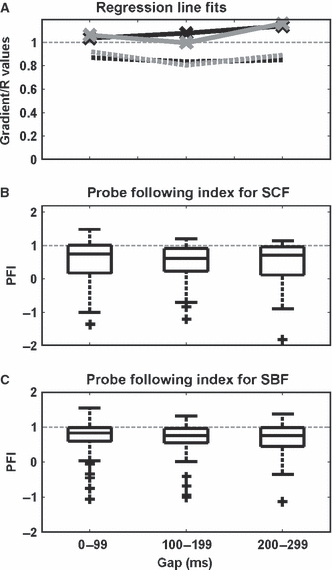
The relationship that the suppressed characteristic frequency (SCF) and suppressed best frequency (SBF) have with the probe tone frequency shows little change with ISI. (A) The gradient of the linear regression for each ISI group for the SCF (black) and SBF (grey). Dashed lines show the correlation coefficients. (B) Box plots showing the probe-following index (PFI) for the SCF for three ISI groups. (C) Box plots showing the PFI for the SBF for three ISI groups.

Figure 6 shows how the locus of suppression depends upon ISI for the population of units (for those conditions when tuning was well estimated, as in Figs 3 and 4). Across the population there was no significant relationship between ISI and the distance from either the SCF to the CF or the SBF to the BF (linear mixed model, *P* > 0.05). The relationship between the probe tone frequency and both the SCF (Fig. 6A; the black line shows the gradient of the regression line calculated in the same way as shown in Fig. 3A, for all ISIs) and the SBF (grey line) remained relatively constant as the ISI was increased. The tightness of these correlations was also relatively constant across time (the dashed lines in Fig. 6A show correlation coefficients for the regression line as shown in Fig. 3A, for all ISIs). This can also be observed in the distributions of the PFI measure, which are shown as box plots in Fig. 6B and C for the SCF and SBF, respectively.

### The threshold and tuning of suppression

In Figs 1 and 2 several examples are shown that suggest that frequency and level of the probe tone not only affect the CF of the forward suppression, but also its bandwidth and threshold. Therefore, we investigated whether there were any systematic differences across the population in the threshold and bandwidth of the suppression as the probe tone frequency and level varied. We applied general linear models (independent variables: the distance of the probe frequency from CF in octaves, the level of the probe relative to the threshold at the probe frequency in the excitatory RF, SCF and ISI) to the data where tuning could be reliably estimated. The models showed that Q_ERB_ was related to the distance (in octaves) of the probe from the CF (*P* < 0.01; but not the level of the probe: *P* = 0.29), and the threshold of suppression was related to the level of the probe relative to the excitatory threshold at that frequency (*P* < 0.01; but not the frequency of the probe: *P* = 0.21).

**Fig. 7:**
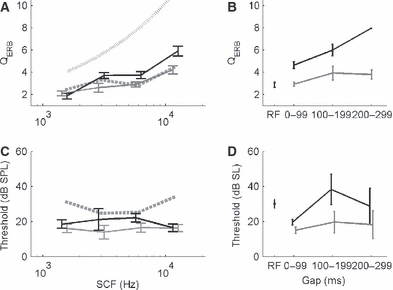
(A) The mean Q_ERB_ of suppressed tuning as a function of suppressed characteristic frequency (SCF) for probe frequencies near to unit CF (< 0.5 octaves away; black line; error bars are standard errors) or off-CF (> 0.5 octaves away; grey line). Also shown are the Q_ERB_ values for the excitatory receptive fields (RFs; coarsely dashed grey line) and for guinea pig auditory nerve fibres (finely dashed line; Evans, 2001). (B) The mean Q_ERB_ of suppressed tuning at different ISIs, for probe frequencies near to CF (< 0.5 octaves away; black line) or off-CF (> 0.5 octaves away; grey line). Also shown is the Q_ERB_ for excitatory RFs (single point). (C) The threshold (conditioner level) of suppression as a function of SCF for ISIs of zero. The black line shows data for probe levels above the 0.4 criterion FTC. The grey line shows data for probe levels below this criterion. The grey dashed line shows the mean excitatory CF thresholds. (D) The threshold of suppression (conditioner-level) as a function of ISI. Also shown are the thresholds for excitatory CFs (single point).

The tuning of suppression became wider as the distance between the probe tone frequency and the CF increased. This is shown for the population data at zero ISI in Fig. 7A, as a function of the SCF. The Q_ERB_ of forward suppression for probes more than half an octave away from the unit excitatory CF (grey) are slightly, but significantly (one-way anova: *P* < 0.01), lower than those nearer to CF (black). Also shown for comparison are the Q_ERB_ values for the excitatory RFs from the same cortical units (grey coarsely-dashed line) and for a function describing the tuning at the level of the auditory nerve (grey finely dashed line; Evans, 1992). This makes it clear that the suppressed tuning is broad relative to the periphery, and more comparable with excitatory RFs regardless of the frequency of the probe. Figure 7B shows the mean Q_ERB_s across the population as ISI varies, when the probe tone frequency is within 0.5 octaves of CF (solid black line) or more than 0.5 octaves from CF (solid grey line). Thus, at all delays, probes placed further away from CF could be suppressed by a wider range of conditioner frequencies.

Figure 7C shows the threshold at SCF, for probes placed either above (black line) or below (grey line) threshold (defined by the 0.4 criterion FTC; all probes produced a significant increase in firing). Probes well within the excitatory RF of the unit tended to produce slightly but significantly (in the general linear model described) higher suppressed thresholds. Notice also that the threshold of suppression was lower than that of excitation (dashed grey line). This indicates that below threshold conditioners could nevertheless have an effect on the response of the probe. Figure 7D shows how the SCF threshold changes with ISI when the probe tone level is higher (solid black line) or lower (solid grey line) than the 0.4 criterion threshold.

These characteristics of threshold and bandwidth most likely relate to recent observations by Scholl *et al.* (2008), who showed that lower level probes result in wider suppression, and other observations that low level probes are more easily masked (Calford & Semple, 1995; Alves-Pinto *et al.*, 2010). In our data, the correlation between the Q_ERB_ and the probe tone level was not significant (*P* = 0.29 in the general linear model above), but the effect of probe level was not investigated systematically. It seems likely that the effect was obscured by the variation of the probe frequency.

### Spike rate measures of frequency specificity

The analysis thus far has examined the locus of tuning with respect to the position of a probe within the excitatory RF, and whether the SCF depended on the probe frequency or the unit CF. An alternative way to consider the data is in terms of the relative reduction in spike counts in a unit for pairs of probe frequencies (f_P1_ and f_P2_), and the conditioner frequencies (f_C1_, f_C2_) when they are matched (f_C1_, f_P1_) or mismatched (f_C2_, f_P1_). If forward suppression is frequency specific then firing rates in response to the probe should be more suppressed when probe and conditioner frequencies are matched.

**Fig. 8:**
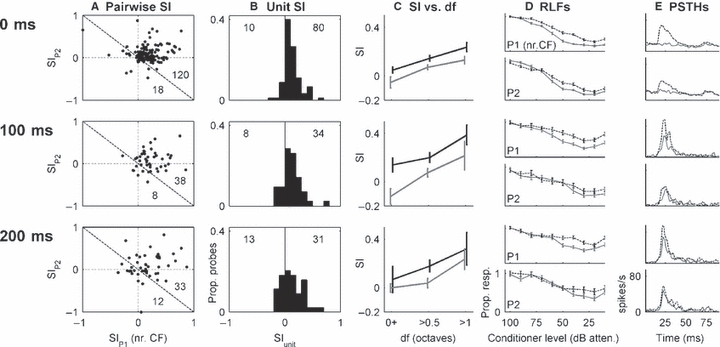
(A) Specificity indexes (SI; Ulanovsky *et al.*, 2003) for the suppression of firing rates for pairs of probe conditions within a unit. The *x*-axis indicates SI for the probe nearest to unit characteristic frequency (CF). Positive values mean suppression is greater when conditioner and probe frequencies are matched. Panels on different rows show data for 0-, 100- and 200-ms ISIs. (B) Unit SIs (overall measure across all probe frequencies in a unit) at different ISIs. (C) Mean SI and SE as a function of the frequency difference (df) between pairs of probes (black lines indicate probe nearest CF). Data are grouped according to df (0 < df ≤ 0.5, 0.5 < df ≤ 1, 1 < df). (D) Mean firing rates (and SE) in response to the probe as a function of conditioner attenuation. Grey lines indicate when probe and conditioner are matched in frequency. P1 panels show rate levels functions for the probe nearest CF, P2 panels show the functions when the probe is the other frequency in the pair. Firing rates are normalized for each unit to the response to the probe alone. (E) Population PSTHs of responses to probe tones, averaged across all conditioner levels louder than 50 dB attenuation. RLF, Rate Level Function.

To examine the effect of matched and mismatched conditioner and probe frequencies, we computed a SI that has been used previously to examine the frequency specificity of adaptation (Ulanovsky *et al.*, 2003, 2004; Malmierca *et al.*, 2009; Anderson *et al.*, 2009; see Materials and methods). Figure 8A, in a plot analogous to those in previous studies, shows the SIs calculated for all probe pairs in which there was a significant response to both probes (at the ISIs indicated). An SI value of greater than zero indicates that the probe was more suppressed by a conditioner at the same matched frequency than by a conditioner at the frequency of the other probe. As there are two probes, there are two SIs. Points lying above the diagonal indicate some bias in favour of matching probes and conditioners. At all delays the population showed a significant number of pairs that were frequency specific (sign test for points lying above the diagonal: *P* < 0.01 at all ISIs). Note also that there are more conditions for which SI_P1_> SI_P2_ (68% overall; sign tests: *P* < 0.05 for 0-ms and 100-ms ISIs, *P* = 0.13 at 200-ms ISI). Because probes were often not symmetrically placed around the unit CF, P1 was always assigned to the probe tone frequency nearest CF. Thus, the bias towards positive values of SI_P1_ indicates that the differential suppression is biased towards probes near to CF. This suggests that although suppression is frequency specific, there is still an effect of CF. Figure 8B shows the summary SI values calculated for each unit, obtained by considering all pair combinations in a unit (see Materials and methods) at a given ISI. Positive values indicate that a unit was on average frequency specific. It shows that the majority of units were frequency specific (sign test: *P* < 0.01 at all delays).

Figure 8C shows the effect of the frequency difference between the probe pairs on the SI for each probe condition. Notice that although most of the points are positive (indicating that suppression is strongest when the conditioner and probe are matched in frequency), the SI values for the probe condition nearest CF (black line) are higher than the probe condition further from CF (grey line). This indicates that suppression is proportionally stronger for probe tones nearer to CF. At the smallest frequency difference the SI is negative for probes further from CF and roughly symmetrical around zero. This would correspond to points that lie on the descending diagonal in Fig. 8A, and indicates a lack of frequency specificity between pairs of probe tones less than half an octave apart.

In Fig. 8A–C, firing rates were summed across all conditioner levels in order to calculate SI values. Figure 8D shows the (normalized) firing rates of the population in response to the probe, for the corresponding pairs of probe and conditioner frequency used in Fig. 8A–C, but this time as a function of conditioner attenuation. At low conditioner levels (large attenuations), conditioners have no effect regardless of conditioner frequency (i.e. the normalized response to the probe is 1). However, at higher conditioner sound levels, the response to the probe is more suppressed when the probe and conditioner are matched in frequency (solid grey lines) than not (dashed black lines). Figure 8E shows corresponding population PSTHs for the probe–conditioner pairs. These were calculated for conditioners at levels of 50 dB attenuation (approximately 50 dB SPL) or louder (including responses at all conditioner levels obscured the small effects seen at longer delays). At short delays matching the conditioner and probe frequency results in almost complete suppression of the probe response. At longer delays this effect becomes more subtle, but the difference between the PSTHs remains significant (Wilcoxon signed ranks test, *P* < 0.01 in all conditions).

This analysis confirms that the effects of frequency specificity in our data are similar to the effects previously reported as changes in firing rates in more extended odd-ball stimulus paradigms. It also shows, as did Figs 3 and 4, that there remains an influence of CF. Suppression caused by conditioner frequencies that are not matched to the probe frequency but that are close to the unit CF can be as strong as the suppression caused by conditioners that exactly match the probe frequency.

### Spike discharge history

In auditory nerve fibres, the response to a probe tone is reduced in direct proportion to the response elicited by the conditioner. Although it is clear that firing rate alone is not a good or complete description of the effect of forward suppression in cortical neurons, a previous study, which looked at context sensitivity for binaural response properties, demonstrated that a majority of units showed significant correlations between the response to the conditioner tone and the decrement in the response to the probe tone, when both were presented at the CF of a neuron (Zhang *et al.*, 2005). We sought to investigate whether there was a change in the dependence of probe tone responses on the response to the conditioner tones (response history) when the probe tone was away from CF.

**Fig. 9:**
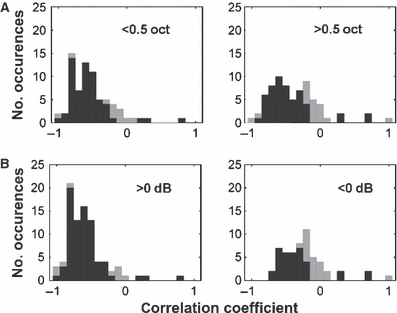
Histograms showing the distribution of Spearman's correlation coefficients where the correlation under consideration is between conditioner and probe tone responses. (A) The left panel shows correlation coefficients for probe conditions in which the probe frequency was within 0.5 octaves of the CF, and the right panel shows correlation coefficients for probe conditions in which the probe frequency was over 0.5 octaves away from the CF. (B) Correlation coefficients for probe conditions in which the probe level relative to the 0.4 criterion RF threshold was above (left panel) and below (right panel) 0 dB re the threshold criterion. The black bars indicate coefficients that were significant, and the open bars indicate coefficients that were not significant.

Spearman's Rho correlation coefficients were calculated for each probe tone condition, from all conditioner–probe combinations in a given probe condition. Probe conditions were then grouped as before depending on the distance from CF or the level of the probe tone. The majority (80%; filled bars) of probe conditions showed a significant effect of discharge history although the range of values was large, consistent with previous reports (Zhang *et al.*, 2005). Figure 9A shows differences in the correlation coefficients when the probe conditions are grouped depending on the distance of the probe from CF. Correlation coefficients were slightly lower for probes that were over 0.5 octaves from the CF (mean: −0.41) than for probe tone frequencies that were within 0.5 octaves of CF (mean: −0.51), and a slightly larger percentage of probe conditions resulted in significant correlations (85%; filled bars) when the probe is close to CF compared with away from it (75%). This trend was consistent with the dependence of tuning on probe frequency, because as probes move away from CF the difference between RF tuning and SRF tuning increases. A larger difference in correlation coefficients is observed when the probe conditions are grouped based on probe level. Figure 9B shows that correlation coefficients are generally larger when the probe level is above the 0.4 criterion threshold (mean: −0.55) than when it is below (mean: −0.29). Also, there are a larger percentage of probe conditions that result in significant correlations when the probe level is above (95%) than when it is below the 0.4 criterion threshold (70%). Thus, the responses to lower level probe tones are overall less related to response history than those that are well within the RF. This is consistent with lower level probes being suppressed by a wider range of stimuli, outside of the unit RF.

Finally, we also compared significant correlation coefficients between probe conditions within units. The effect of probe condition on the correlation coefficient was small (median difference: 0.11) compared with the distribution across the population. Thus, the degree to which discharge history can account for forward suppression characteristics appears to depend more on the unit than on the choice of probe. It may also reflect a substantial overlap between SRF and RFs across all probe conditions tested in a unit. If an RF and SRF do not overlap at all, the correlation between conditioner and probe responses must be near zero. However, large SCF changes may occur with small changes in the degree of overlap between SR and SRF, which might result in only small changes in the correlation between conditioner and probe firing rates.

## Discussion

In a two-tone forward suppression paradigm, tuning of the suppression in auditory cortex was most often centred near to the probe tone frequency. Suppression was frequency specific at all ISIs tested (up to 300 ms). Tuning was more closely related to the probe frequency, and less influenced by unit CF, when the probe was well away from CF. Off-CF probes also produced more broadly tuned suppression.

The main effect of the shift in tuning observed when the probe tone was away from CF was consistent with that reported previously in a small sample (Calford & Semple, 1995). That study suggested that suppressed tuning was ‘biased towards’ the probe frequency, rather than centred on it. The size of the effect of the probe frequency varied in our data, which showed a tendency for suppression to be determined by CF for probes near to (but not at) CF and by the probe frequency when this was well away from CF. In most other respects, such as the dependence of tuning and thresholds on ISI, our data are similar to forward suppression data when the probe tone was predominantly presented at CF (Calford & Semple, 1995; Brosch & Schreiner, 1997). Previous studies in the cortex (Brosch *et al.*, 1999; Brosch & Schreiner, 2000; Brosch & Scheich, 2008) have also reported a dependence of enhancement on probe tone conditions, and found that probe tones remote from CF produced less well-defined patterns of enhancement. Consistent with this, we rarely observed enhancement of the probe tone response and, in our data, it was difficult to differentiate such enhancement from late responses to the conditioner tones. Enhancement is less common at very short ISIs, and in a given unit occurs over a short range of ISIs (Brosch & Schreiner, 2000). Therefore, our fairly sparse sampling of other ISIs made observation of enhancement less likely.

Scholl *et al.* (2008) recently found that forward suppression had a larger bandwidth for probe tones at lower sound levels. Our data do not show a significant effect of probe level on bandwidth. Instead, when probe tones are further from CF, suppressed tuning curves broaden, whilst responses to lower level probe tones are more easily suppressed by low-level conditioner tones. Because we did not vary probe level independently of frequency, it seems likely that the effect of level on bandwidth was diluted. In general, signals that elicit less activity are more easily suppressed (Calford & Semple, 1995; Alves-Pinto *et al.*, 2010). Data on binaural interactions between two tones, similarly, show that lower level probe tones are suppressed by a wider range of binaural levels (Zhang *et al.*, 2005).

Other cortical studies have examined longer sequences of longer duration tones. McKenna *et al.* (1989) reported effects of order, both facilitation and suppression, for sequences of five tones of different frequencies. Highly frequency-specific habituation was reported (Condon & Weinberger, 1991) following several minutes of repeated stimulation at one frequency. There is also evidence of frequency-specific sequential interactions in spectrotemporal RFs, derived from random-chord style stimuli (Pienkowski & Eggermont, 2010). Adaptation that was specific to the stimulus frequency has been reported for long sequences (e.g. stimuli consisting of 400 tones of 230 ms duration with an ISI of 506 ms; Ulanovsky *et al.*, 2003, 2004) where the probability of different stimulus frequencies was manipulated. A substantial proportion of this frequency-specific adaptation was a local effect between adjacent tone pairs. Our data show that forward suppression is specific to the stimulation frequency even at timescales of a few tens of milliseconds. Ulanovsky *et al.* (2003) also reported that the tuning of the adaptation they observed resulted in ‘hyperacuity’: very high sensitivity to frequency differences between tones. In contrast, the bandwidths of forward suppression in our data were on average much broader than that of peripheral tuning. It may be that ‘hyperacuity’ only occurs at longer ISIs, developing with sequences of tones or longer duration tones (see Brosch & Schreiner, 1997, fig. 13).

Dependence of suppression on probe-tone frequency and level has also been observed in two-tone paradigms in the IC. Malone & Semple (2001; Fig. 10) observed forward suppression centred on the probe-tone frequency, in at least one unit, using longer tone durations. However, most of their probe tones were placed at CF. A preliminary report of a recent more systematic investigation (Stakhovskaya *et al.*, 2008) suggested that at rostral locations within the IC, forward suppression tuning at short ISIs is affected by the frequency of the probe tone, whilst at caudal locations suppression is determined by the excitatory RF. Brimijoin & O'Neil (2010) have also reported frequency-specific forward suppression in the IC of the moustached bats. Thus, frequency-specific interactions are evident at various levels of the auditory system, although it is currently unclear how the responses in IC and A1 compare quantitatively. Frequency-specific adaptation has also been shown subcortically using sequences of tones (Perez-Gonzalez *et al.*, 2005; Anderson *et al.*, 2009; Malmierca *et al.*, 2009). These effects are more prominent in the non-lemniscal auditory pathway, such as the dorsal cortex of IC (Malmierca *et al.*, 2009) or the medial geniculate body (Anderson *et al.*, 2009), than in the core lemniscal pathway. In general, frequency-specific adaptation is observed at higher rates of presentation in IC than in A1 (Ulanovsky *et al.*, 2003; Malmierca *et al.*, 2009).

Several other lines of evidence suggest that frequency specificity in the cortex is only partly inherited from lower nuclei. Current evidence suggests that frequency specificity is actually stronger in IC (Malmierca *et al.*, 2009) than in the thalamus (Anderson *et al.*, 2009), posing a problem for any simple hypothesis of inheritance. Additionally, frequency-specific adaptation of local field potentials in A1 increases for recording sites further away from the main input from the thalamus (Szymanski *et al.*, 2009). In our data, frequency-specific interactions are clear at ISIs of 200 ms, which are difficult to attribute to subcortical forward suppression (Schreiner, 1981). Also, the bandwidths of frequency-specific suppression in our data are much broader than peripheral tuning (see Evans *et al.*, 1992; or in the brainstem, Sayles & Winter, 2010), suggesting that a considerable degree of across-frequency convergence precedes the two-tone frequency specificity we report. It is thus likely that the frequency-specific adaptation observed in cortical neurons is further influenced by the convergence of both thalamo- and cortico-cortical inputs.

The underlying mechanisms of cortical forward suppression remain unclear. A logical hypothesis to explain frequency-specific adaptation is that adaptation is occurring at or before the inputs to the recorded neuron. If these inputs have different tuning properties, then forward suppression of different probe frequencies will reflect the suppression of different inputs. Frequency-specific forward suppression may be mediated by γ-aminobutyric acid (GABA) ergic inhibition (Calford & Semple, 1995; Brosch & Schreiner, 1997). Eytan *et al.* (2003) have demonstrated a GABAergic-dependent stimulus-specific suppression and enhancement *in vitro*. However, intracellular recordings have shown that inhibitory synaptic conductances elicited by a brief sound last up to 100 ms, while suppression of the response to a successive sound could last 500 ms or longer (Wehr & Zador, 2005). We found no difference in the tendency for the locus of suppressed tuning to follow the probe frequency for ISIs above and below 100 ms. One interpretation of this is that synaptic depression and inhibition therefore both exhibit frequency specificity. If indeed synaptic depression dominates the forward suppression effect at gaps of more than 100 ms, then evidence from our data and other studies suggests that synaptic depression is frequency specific. Our observations of similar characteristics at short timescales might suggest that inhibition shares this property (as in Eytan *et al.*, 2003). However, it might equally be true that the effect of probe condition we see at zero ISIs nevertheless reflects synaptic depression, which is evident despite the presence of non-specific inhibition. Denham & Winkler (2006) have proposed a model of convergent inputs to a cortical neuron, all of which have depressing synapses, which our data appear to be consistent with. In this model, different probe frequencies would preferentially drive inputs with different tuning characteristics and those synapses would be maximally depressed by conditioner tone frequencies close to the probe tone. A similar model has been shown to explain some of the differences in spectrotemporal RFs derived from different stimuli (David *et al.*, 2009).

In summary, we have found that the tuning of cortical forward suppression is more consistent with the frequency of the probe than with the excitatory tuning of a neuron. Thus, adaptation in the auditory cortex is ‘frequency specific’ for rapidly presented isolated pairs of tones. Nevertheless, influence of unit CF remains strong when the probe is close to CF. Our data are consistent with the hypothesis that forward suppression in individual cortical units occurs to some degree independently within different processing channels. At very short timescales, these channels have different but broadly tuned and overlapping frequency ranges.

## Abbreviations

A1: primary auditory cortex
BF: best frequency
CF: characteristic frequency
ERB: equivalent rectangular bandwidths
FTC: frequency threshold curve
GABA: γ-aminobutyric acid
IC: inferior colliculus
ISI: interstimulus interval
MU: multi-unit
PFI: probe-following index
SBF: suppressed best frequency
SCF: suppressed characteristic frequency
SFTC: suppressed frequency threshold curve
SI: specificity index
SR: spontaneous rate
SRF: suppressed receptive field
SU: single unit
